# A 21 m Operation Range RFID Tag for “Pick to Light” Applications with a Photovoltaic Harvester

**DOI:** 10.3390/mi11111013

**Published:** 2020-11-18

**Authors:** Aingeru Astigarraga, Alberto Lopez-Gasso, Diego Golpe, Andoni Beriain, Hector Solar, David del Rio, Roc Berenguer

**Affiliations:** 1 TECNUN—Technological Campus of the University of Navarra, Paseo de Manuel Lardizabal, 13, 20018 San Sebastian, Spain; a905254@alumni.unav.es (A.A.); algasso@tecnun.es (A.L.-G.); dgolpe@tecnun.es (D.G.); aberiain@tecnun.es (A.B.); hsolar@tecnun.es (H.S.); ddelrio@ceit.es (D.d.R.); 2 Ceit–Basque Research and Technology Alliance, Paseo de Manuel Lardizabal, 15, 20018 San Sebastian, Spain

**Keywords:** RFID tag, voltage limiter, RFID reader, photovoltaic harvester, “pick to light”

## Abstract

In this paper, a novel Radio-Frequency Identification (RFID) tag for “pick to light” applications is presented. The proposed tag architecture shows the implementation of a novel voltage limiter and a supply voltage (VDD) monitoring circuit to guarantee a correct operation between the tag and the reader for the “pick to light” application. The feasibility to power the tag with different photovoltaic cells is also analyzed, showing the influence of the illuminance level (lx), type of source light (fluorescent, LED or halogen) and type of photovoltaic cell (photodiode or solar cell) on the amount of harvested energy. Measurements show that the photodiodes present a power per unit package area for low illuminance levels (500 lx) of around 0.08 μW/mm^2^, which is slightly higher than the measured one for a solar cell of 0.06 μW/mm^2^. However, solar cells present a more compact design for the same absolute harvested power due to the large number of required photodiodes in parallel. Finally, an RFID tag prototype for “pick to light” applications is implemented, showing an operation range of 3.7 m in fully passive mode. This operation range can be significantly increased to 21 m when the tag is powered by a solar cell with an illuminance level as low as 100 lx and a halogen bulb as source light.

## 1. Introduction

Internet of Things (IoT) technology seeks to connect numerous devices and objects to a global network, sharing data that are collected with built-in sensors. The need to connect a large number of sensors to a network has aroused great interest in reducing the production and maintenance cost of such devices, without compromising on either autonomy or communication range. Taking these interests into account, passive RFID sensors have proved to be a promising technology for IoT [1,2,3]. In passive RFID sensor systems, a reader wirelessly supplies energy to a tag. The tag uses this energy to demodulate the incoming information and power up all the loads connected to it. Finally, the tag responds by sending information such as the Identification (ID) or sensor data, using backscattering to modulate the returning signal. RFID systems by themselves are widely used identification and tracking technologies in many industries, but combining the data acquired by sensors with RFID data further expands the applications of these devices. Besides that, since passive RFID systems do not require a battery, these sensors are low cost and have an enhanced autonomy.

Despite the previously mentioned benefits, this fully passive system’s main disadvantage is the need to be close enough to the reader, so that it can supply the necessary power to the tag. This limits the communication range of the tag, reducing the number of potential applications. One solution that has been pursued is using battery-assisted passive (BAP) RFID tags [4,5]. Using a battery as the main power supply considerably improves the communication range, as it is now limited by the quality of the communication link instead of the tag’s power requirements. However, this alternative increases the maintenance cost of the device, as well as its environmental impact. To avoid this tradeoff, studying the use of other energy sources besides batteries, such as kinetic, thermal or solar energy, has become an interesting field of study [6,7,8,9,10,11,12]. Nevertheless, the implementation of a small-sized energy harvester that meets the power requirements of an RFID sensor tag poses multiple challenges. This is especially true when considering that the harvested energy is subject to large variations, which could result in a less reliable power supply when compared to a battery.

**Figure 1:**
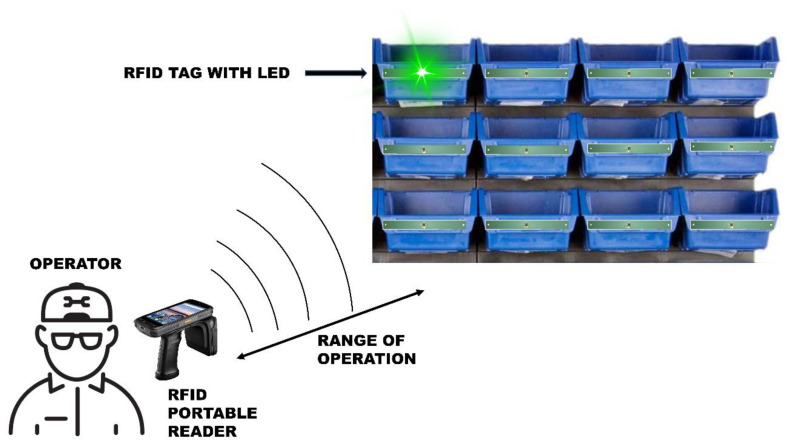
Example of an Ultra High Frequency (UHF) RFID-based “pick to light” system.

This paper studies the use of photovoltaic energy harvesting techniques, with a focus on indoor lighting environments, to extend the communication range of a battery-less RFID “pick to light” tag. Using this method, depending on the lighting conditions, the communication range can be improved over a fully passive system and even reach the values of a BAP system. The tag architecture, which can operate both in fully passive and BAP mode, is presented and used in an indoor “pick to light” system, like the one shown in Figure 1. Therefore, the feasibility of the considered energy harvesting method is analyzed by comparing it with the performance of the two main operation modes: fully passive and using photovoltaic energy as a supply source.

The paper is organized as follows. In Section 2, various light harvesting devices are analyzed as potential choices to supply power to the tag, and a description of the RFID tag’s architecture and modes of operation is given. Measurement results of the photovoltaic cell powered RFID “pick to light” tag are presented in Section 3. Finally, relevant conclusions are provided in Section 4.

## 2. Solar-Powered Semi-Passive RFID Tag

Photovoltaic energy harvesting can be a great alternative to batteries for BAP RFIDs. In the presence of the right lighting conditions, this harvesting method can provide the tag with the necessary power to operate, even when the reader is far away from the tag. Therefore, it is crucial to know first under which type of lighting conditions the RFID is going to operate, as this will narrow the range of photovoltaic harvesting devices that can be used for this purpose.

### 2.1. Lighting Conditions and Harvester Choices

**Figure 2:**
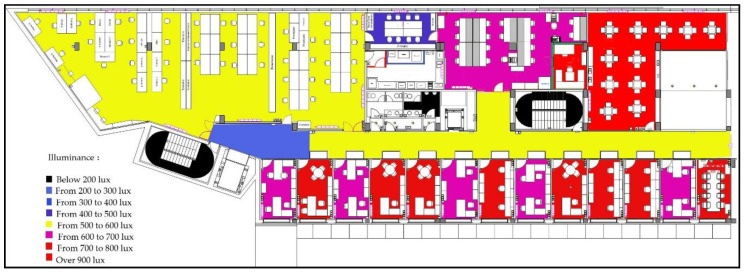
Illuminance level mapping of a research institute building’s floor.

This study focuses on the use of a semi-passive RFID tag in indoor environments. One way to characterize the lighting conditions is the illuminance level, which indicates the number of lumens per square meter (lux, lx). This is the typical criteria to fix a reference level for luminance level in different indoor environments. For indoors, these levels can go from as low as 100 lx in areas such as corridors or storage spaces, up to a few thousands of lux for rooms where highly detailed tasks are performed [13]. Figure 2 shows the measured average illuminance levels for the different rooms of an entire building floor (offices, laboratories, storage rooms, etc.), which were measured with a digital light meter [14]. The map shows multiple workplaces and small rooms, which are illuminated with artificial light, sunlight, or a mixture of both. By looking at the illuminance levels, it is interesting to notice that most rooms manifested an average illuminance above 500 lx.

**Figure 3:**
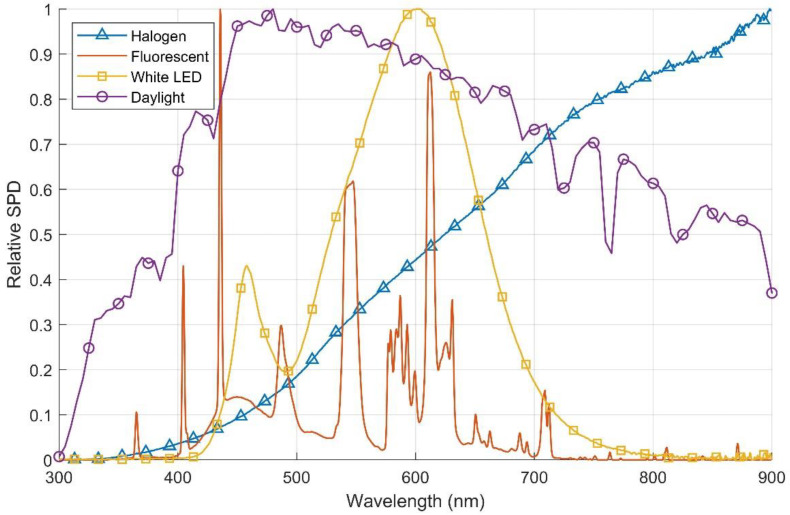
Relative spectral power distribution of some common light sources.

Despite the importance of illuminance, it is also crucial to consider the spectral power distribution (SPD) of the light source, which indicates the emitted power as a function of the wavelength. Some light sources might display a considerable power only in a small bandwidth, while others such as sunlight have great power across a wide bandwidth, partially outside the visible spectrum defined by the luminance measurement. To better illustrate this phenomenon, the relative SPDs of some common light sources are shown in Figure 3 [15,16]. As can be observed, LEDs and fluorescents emit power mostly in the visible spectrum (380–750 nm), and even in that spectrum they display a narrow bandwidth. On the other hand, halogen sources emit low power in the lower end of the visible spectrum, but the power increases with the wavelength up to part of the infrared spectrum. Finally, the power of daylight is quite homogeneously spread compared to the other sources, reaching its peak close to 500 nm. Overall, it can be concluded that for the same illuminance levels, using photovoltaic harvesters with equal efficiency across the full spectrum shown in Figure 3, daylight would generate the most power, followed by halogen bulbs, LEDs and finally fluorescents.

**Figure 4:**
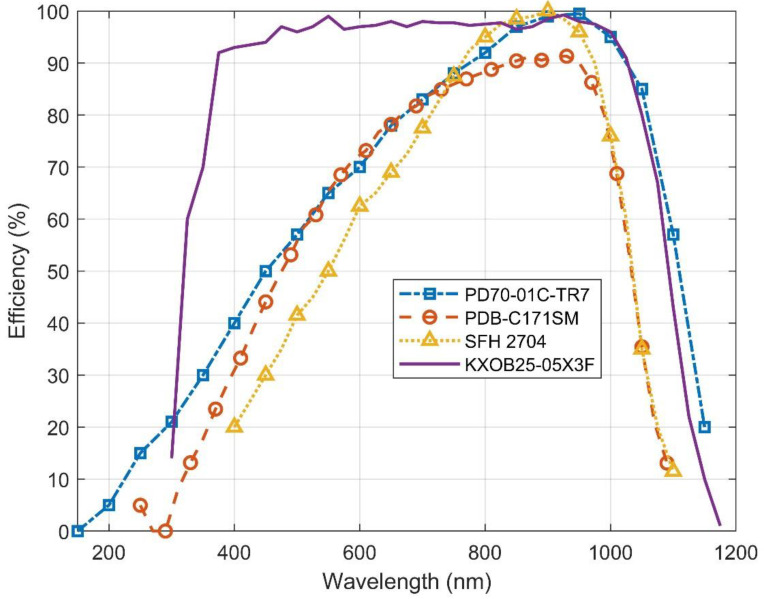
Quantum efficiency of the PD70-01C-TR7, PDB-C171SM and SFH 2704 photodiodes, as well as the KXOB25-05X3F solar cell.

It is also important to notice that the conversion efficiency of photovoltaic harvesters varies with wavelength. Therefore, it is necessary to consider not only the light source from which the energy will be harvested, but also the harvesting device that has an acceptable efficiency across that emission spectrum. Figure 4 shows the quantum efficiency of various photovoltaic energy harvesters [17,18,19,20].

Two different types of photovoltaic energy harvesting devices were considered: photodiodes and solar cells. Table 1 presents the different measured devices using a fluorescent lamp as a light source for the 500 lx measurements and sunlight for the 22 klx measurements (the measurement setup is introduced in Section 3.1). As can be observed, the SFH 2704 and PDB-C171SM photodiodes present a power per unit package area for low illuminance levels (500 lx) of 0.08 and 0.06 μW/mm^2^, respectively, which are slightly higher and equal to that measured for the IXYS KXOB25-05X3F solar cell, which presents a power per unit package area of 0.06 μW/mm^2^. However, the power-to-area ratio of the photodiodes does not increase as much as that of the solar cell when the illuminance level rises. As shown in Table 1, the power per unit package area at 22 klx is 4.17 μW/mm^2^ for the SFH 2704 photodiode, while for the IXYS KXOB25-05X3F solar cell, it is 6.12 μW/mm^2^. It is also important to notice that the peak power at 500 lx generated by one SFH 2704 photodiode, which is the one with the highest power per unit package area, is only 0.3 μW. This is because the photodiode is not designed with the purpose to generate energy and, therefore, it is not easy to find implementations “off the shelf” with a large number of diodes in parallel. As a theoretical exercise, for an expected power consumption from the tag + LED of around 31 μW (see Section 2.3), around 100 photodiodes in parallel would be needed to obtain the required output power. In addition, in contrast with photodiodes, the KXOB25-05X3F displays a high efficiency across a large spectrum bandwidth, as previously shown in Figure 4. This gives the solar cell more versatility, as is it will be able to harvest energy from a broader range of light sources and provide a larger output power when a wideband source is employed. Hence, the KXOB25-05X3F was chosen to power the semi-passive RFID with pick to light capability.

Therefore, when considering a photovoltaic harvesting system, not only the environment illuminance level should be considered but also the light source type and the photovoltaic harvester device efficiency across the spectrum bandwidth for optimum performance.

### 2.2. RFID Tag Architecture

**Figure 5:**
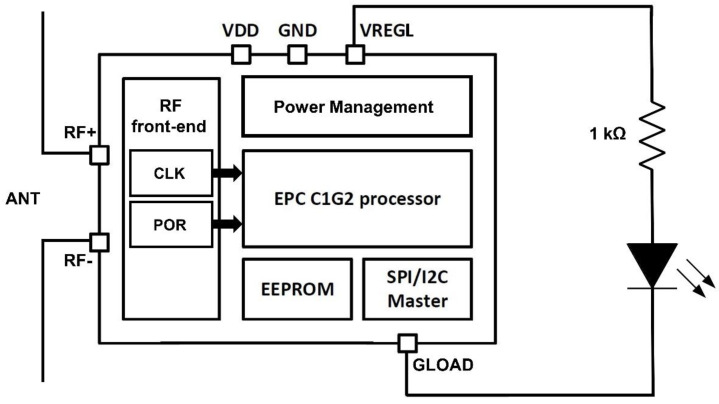
Block diagram of the RFID tag connected to the LED.

The proposed “pick to light” RFID tag is presented in Figure 5. It consists of a commercial RFID tag [23,24] connected to a dipole antenna with 2.1 dBi gain, which controls and supplies voltage to a surface mount LED [25].

The RFID includes the standard modules: the RF front-end, the power supply management unit and the digital processor that supports the EPC C1G2 standard amongst others. The RF front-end demodulates the incoming Amplitude Shift Keying (ASK) modulated signal from the reader, modulates the backscattered response of the tag to the reader and harvests additional energy from the incoming RF signal. It also provides a reference clock to the digital processor, and it is in charge of enabling the other blocks of the tag once enough energy is available. Finally, the digital processor establishes the communication protocol between the tag and the reader, and it detects when a certain command is received from the reader to enable the LED.

Considering the specific “pick to light” application, the power management unit of the transponder includes a digitally configurable Low Drop Ouput (LDO) regulator controllable by the digital processor to supply power to the connected loads through the VREGL pin [23]. This way, when a specific tag with a particular ID receives the command to blink, the LDO output (VREGL) is enabled. The LED is connected to VREGL output using a 1 kΩ resistor in series to reduce the current flowing through the LED, while proper lighting is assured.

The minimum operation voltage of the LED (2.5 V) is higher than the RFID chip’s minimum operation voltage (1.2 V). Therefore, the chip may be enabled, and it communicates with the reader without having enough voltage to make the LED blink. At that point, enabling the output LDO would be counterproductive as it would sink power without any blink. To avoid this, the pick to light system makes use of a configurable integrated VDD voltage monitor that the RFID chip incorporates [23]. This way, during the initial communication between the reader and the tag, the tag acknowledges the reader whether it has enough energy (a minimum voltage of 3 V stored in the 100 nF supply capacitor connected to the VDD pin) to perform a set of blinks. This way, the reader sends the blinking order to the selected tag only when the voltage condition is met, leading to a more efficient available power management.

The solar cell (Section 2.3) is in charge of supplying voltage and power directly to the tag through the externally accessible VDD pin of the chip. As in every energy-harvesting device, the solar cell has the problem of large available output power under certain external conditions. As an example, if there is a tag supplied by a photovoltaic cell exposed to sunlight, the amount of harvested power will be higher than the one sunk by the tag, provoking rises in the VDD pin voltage above the maximum 3.3 V allowed by the chip. In order to protect the circuit from these voltage peaks, the RFID chip should incorporate a DC voltage limiter in the VDD pin and not in the RF input as some RFID solutions have [26].

**Figure 6:**
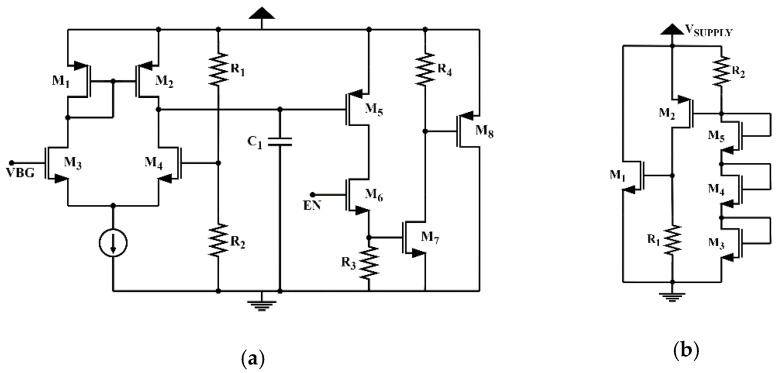
Schematics of the RFID tag’s voltage limiters: (**a**) high accuracy voltage limiter; (**b**) fast response voltage limiter.

The Voltage Limiter (VL) implemented in the selected transponder is reported in [23,27]. It controls the maximum voltage in VDD pin by sinking the excessive available power on it. In contrast to conventional VLs, it uses a bandgap voltage (VGB) as a voltage reference to control the maximum allowable voltage (Figure 6a). Since the designed bandgap circuit generates a precise voltage reference across Process Variations and Temperature (PVTs), the limiting value of the supply voltage experiences very little voltage variations in the presence of different amounts of harvested power, protecting the tag IC. The major drawback of this architecture is its slow response, due to the settling time of the bandgap voltage when the VDD pin voltage rises from 0 V. If there is a fast rise in the VDD pin voltage and the voltage limiter does not respond quickly enough, it might damage the tag IC. Because of that, the RFID tag also incorporates a self-referenced and fast-response VL shown in Figure 6b. Once the bandgap voltage is settled, the VL in Figure 6b is disabled and the one in Figure 6a is enabled [28].

### 2.3. Solar Cell Implementation

**Figure 7:**
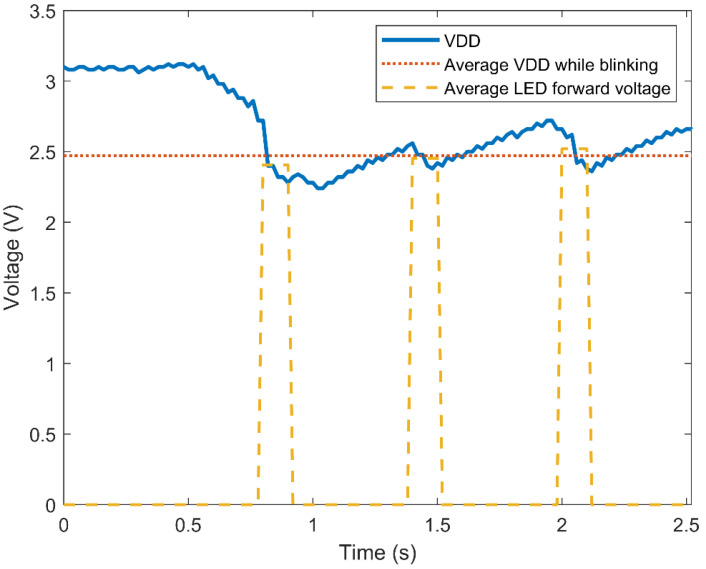
Supply voltage of the RFID tag while the LED is blinking.

**Figure 8:**
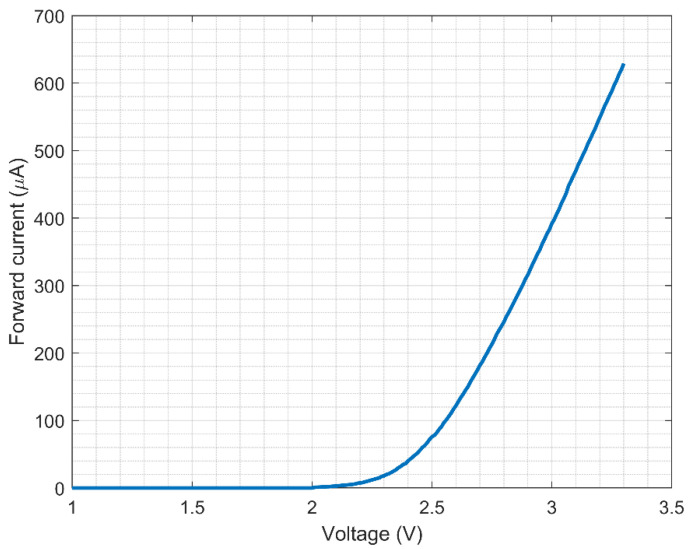
Measured forward current of the surface mount LED [25] with a resistor of 1 kΩ added in series, as a function of the VREGL pin voltage.

To combine the solar cell with the “pick to light” tag, first, an analysis of the required power consumption was conducted. This way, the photovoltaic harvester’s output power requirements can be drawn. Two operation modes can be distinguished in the tag: the first mode corresponds to the typical RFID tag operation, when the communication with the reader is established. This mode typically requires a minimum voltage in the VDD pin of 1.2 V and an available power of around 8 μW [23]. The second mode corresponds to the LED blinking. Figure 7 shows the measured voltage of the VDD pin, while the LED blinks three times in a 1.3-s span. As can be observed, the duration of the control signal (average LED forward voltage) is 100 ms high and 500 ms low, and three pulses are generated by the digital block. After the third pulse, a low signal of 1.9 s is set by the digital block of the tag. In addition, the measured LED’s forward current as a function of the VREGL pin voltage is presented in Figure 8 with a resistor of 1 kΩ added in series like in the tag implementation. Using Figure 7 and Figure 8, an average voltage of 2.5 V in the VDD pin with an average power consumption of 31 μW is estimated for a successful blinking using the previous lighting control pattern. Considering that blinking is the most demanding mode and that the KXOB25-05X3F solar cell has a maximum output power of 11.57 μW at 500 lx (as shown in Table 1), no less than three solar cells would be needed in the implementation.

**Figure 9:**
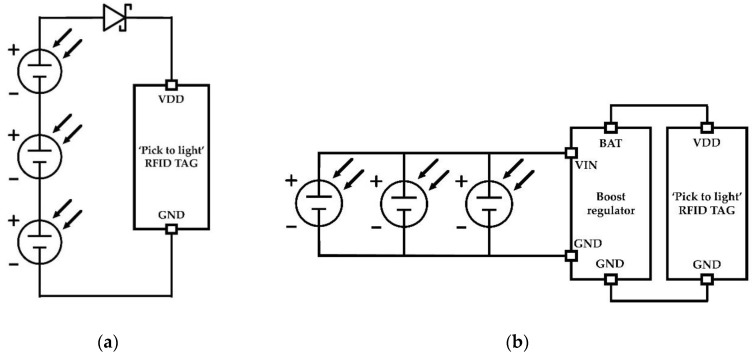
Proposed arrangements to supply the “pick to light” tag with the IXYS KXOB25-05X3F photovoltaic cell: (**a**) three photovoltaic (PV) cells in series with a Schottky diode; (**b**) three photovoltaic cells in parallel with a boost regulator.

Two different arrangements for the solar cells are proposed (Figure 9). The first one connects three solar cells in series, directly to the VDD pin of the RFID tag through a Schottky diode [29]. This diode has a low built-in potential (0.2 V) and it is necessary to avoid any leakage currents that could discharge the tag’s internal capacitor if an object suddenly covers the photovoltaic cell or the energy harvested from the RF interface is higher than the one harvested from the photovoltaic cell. This setup provides three times the output voltage of a single solar cell, with the same output current. Therefore, it maximizes the generated output voltage. An equivalent power could also be obtained if three solar cells were connected in parallel instead, but the output voltage obtained for indoor illuminance levels would be lower than the minimum operation voltage of the RFID tag Integrated Circuit (IC) [23]. The second approach connects three solar cells in parallel, into a boost regulator [30] which connects its output to the VDD pin of the tag. The reason for using three cells in parallel is because the boost regulator can exit the cold startup faster if a larger input current is provided. With this arrangement, once the cold startup ends, a stable supply voltage is obtained.

The two arrangements shown in Figure 9 have different advantages and disadvantages, depending on the application they will be used for. As will be discussed in the following sections, the setup in Figure 9a is simpler and has a high output voltage even for low illuminance levels, while the arrangement of Figure 9b has a more stable output voltage due to the boost regulator but needs some time to reach the desired output level.

## 3. Measurement Results

To better understand and check the performance improvements of the semi-passive RFID tag powered by a photovoltaic cell, multiple types of measurements were conducted. These measurements characterize the performance of the two arrangements proposed at the end of Section 2.3, both with and without the tag. Different types of light sources are also considered.

### 3.1. Solar Cell Arrangement

**Figure 10:**
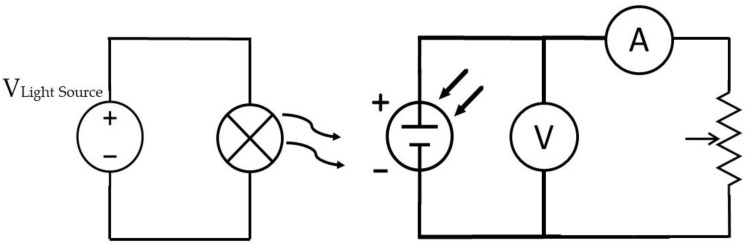
Measurement setup for solar cell characterization.

The first solar cell setup that was considered to power the RFID “pick to light” was to connect three KXOB25-05X3F cells in series. To better understand the behavior of the solar cells, these devices’ electrical characteristics were measured for different illuminance levels and lighting conditions. The measurement setup is presented in Figure 10. As shown, the solar cell was illuminated with a light source, whose light level can be adjusted until obtaining the desired illuminance value with the help of a digital light meter [14]. Then, both the output current and voltage of the solar cells were measured for different load values, which were controlled with a potentiometer.

**Figure 11:**
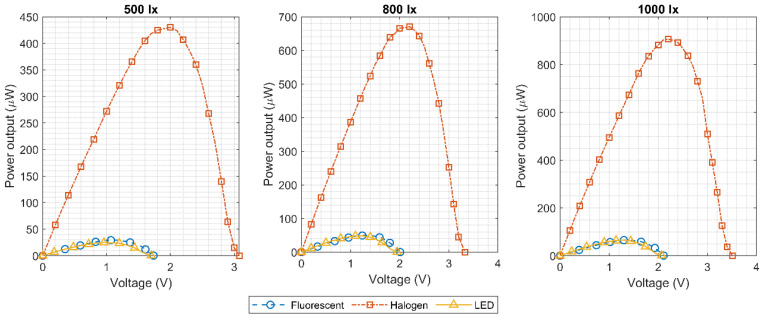
Measured output power of three KXOB25-05X3F solar cells in series.

**Figure 12:**
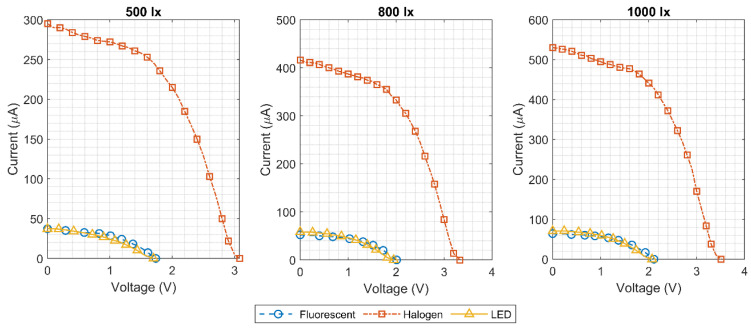
Measured I-V curves of three KXOB25-05X3F solar cells in series.

This setup was used in three different lighting conditions: LED (23 W), fluorescent (15 W) and halogen (50 W). The illuminance levels for the measurements were 500, 800 and 1000 lx, which are typical values for an indoor environment as previously shown in Figure 2. Figure 11 shows the measured output power curve of the three cells in series arrangement, while the measured I–V curves are displayed in Figure 12. The LED and fluorescent lightbulbs achieved similar performances, characterized by a sufficient maximum power, but a poor open circuit voltage. On the other hand, as expected from the initial analysis in Section 2.1, the output power of the solar cells was much greater, and the open circuit voltage was significantly larger when they were illuminated with a halogen light bulb. As explained before, the luminance level which characterizes the spectral power within the visible spectrum (380–750 nm) does not consider the energy in the infrared region. Halogen bulbs, as explained in Section 2.1, present a large amount of energy in the infrared region at the expense of higher power consumption when compared to fluorescents or LEDs. This energy in the infrared region is effectively harvested by the solar cell generating higher output power and open circuit voltage.

### 3.2. Photovoltaic Cell Powered Tag

Once the solar cells were characterized, they were implemented in a semi-passive RFID system. For these measurements, the solar cell was connected to the VDD pin of the tag via a Schottky diode, as explained before, to avoid any leakage currents that could discharge the tag’s internal capacitor. This means that the tag will be powered by the solar cells when there is enough light energy, but will still be able to rely on the integrated RF harvester when the output voltage of the solar cells is lower than that at the output of the voltage multiplier implemented in the RFID tag.

**Figure 13:**
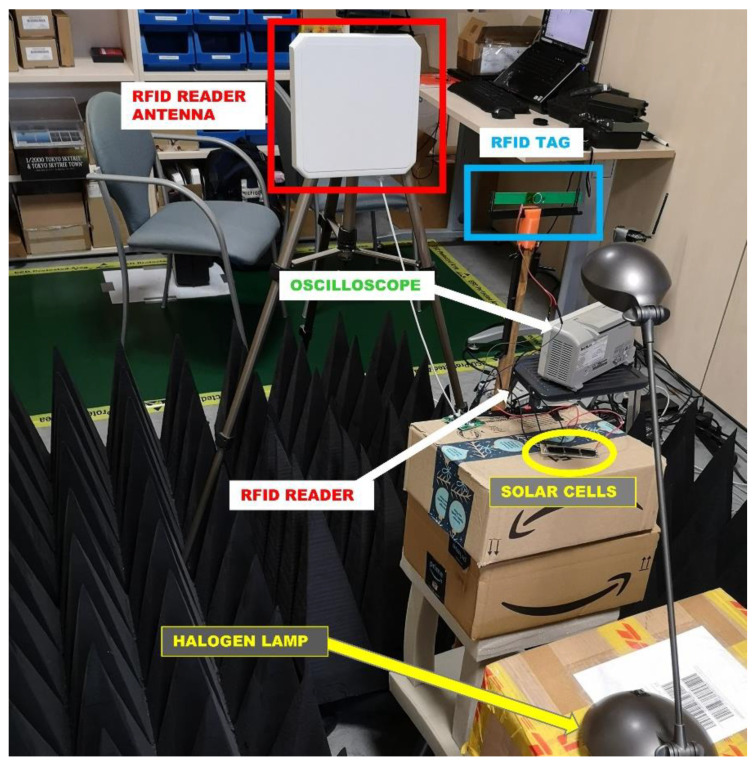
Measurement setup of the semi-passive RFID “pick to light” tag.

The measurement setup for the proposed system is presented in Figure 13. The solar cells were lighted by a halogen lamp and connected to the RFID tag, which was mounted on a stand. In front of the tag, at a distance of 70 cm, an 8.5 dBi antenna with circular polarity [31] was mounted on a tripod. The antenna was connected to an 868 MHz RFID reader [32] through a 10 dB attenuator, to compensate for the limited measurement space. The output power of the RFID reader could be controlled from 10 dBm to 31.5 dBm with steps of 0.25 dB. In addition, an oscilloscope was used to monitor the supply voltage of the tag. To provide some protection against reflections, pyramid-shaped radiation-absorbent foam was placed on the floor and the ceiling. However, it should be noted that this setup is still vulnerable against side reflections, which could slightly alter the measurement results due to constructive or destructive interferences, but these are minimized by placing the reader antenna and the tag at 70 cm from each other.

With this setup, both the reading range and blinking range of the tag were measured, when the “pick to light” tag was operating in a fully passive mode. The adopted procedure to calculate the reading range is as follows:

1. A commercial reference tag was placed in the measurement set-up at a fixed distance of 70 cm between the reader and tag.
2. The output power of the reader was gradually increased until a successful communication was reached.
3. Considering the reader output power (*P_TX_*), its antenna gain (*G_TX_*), the reference RFID chip sensitivity (*P_sens_*), its antenna gain (*G_RX_*) and the wavelength, the theoretical communication range (*d*) was calculated using Friis equation [33]:

(1) $$d=\sqrt{\frac{P_{TX}G_{TX}G_{RX}}{P_{sens}}}\frac{\lambda }{4\pi }$$

4. A correction factor was applied to the reader output power to compensate for the environmental effects and adjust the calculated distance to the real distance of 70 cm.
5. Steps 1 and 2 were repeated with the tag under test in the measurement set-up.
6. Using the reader output power obtained in step 5 and the correction factor of step 4, the tag sensitivity was obtained using Friis equation.
7. Considering that according to the European regulation, the reader can transmit with up to 2 W EIRP and the tag sensitivity, the maximum communication range was calculated.

The measured sensitivity for the tag ID reading was −14 dBm, while the sensitivity to make the tag blink was approximately −9 dBm. This result was expected since, as mentioned in Section 2.3, the most power-demanding function of the pick to light application is to make the LED of the tag blink. The minimum power consumption estimated for each task was 8 μW to read the tag ID and 31 μW to blink the LED. The measured sensitivities correspond to a tag range of approximately 5.9 and 3.7 m, respectively.

**Figure 14:**
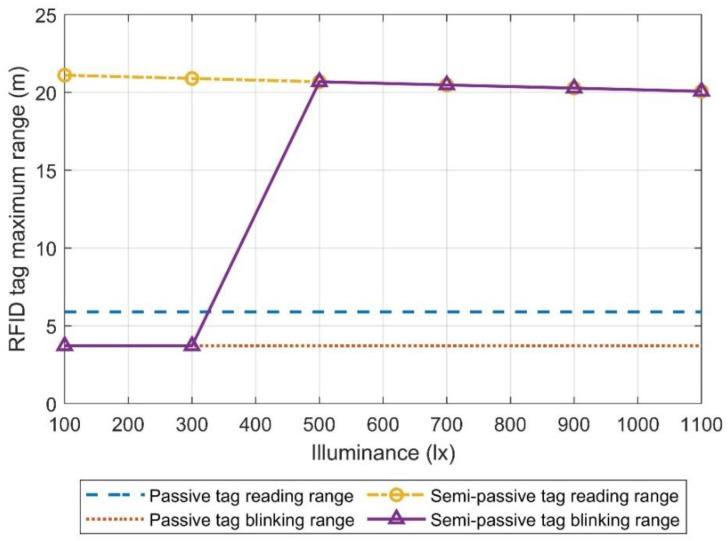
Maximum tag ID reading range and LED blinking range of the proposed semi-passive RFID tag, as a function of illuminance.

Figure 14 shows the RFID tag’s maximum communication ranges when it was powered using the first solar cell configuration, where three solar cells in series were directly connected to the VDD pin of the RFID tag through a Schottky diode. The light source was a halogen bulb. As shown, noticeable improvements in the maximum range of the tag were observed. For the tag ID reading range, the maximum range increased from 5.9 to 21 m at an illuminance level of only 100 lx. On the other hand, the blinking range only obtained a substantial upgrade for illuminance values above 500 lx, approximately.

**Figure 15:**
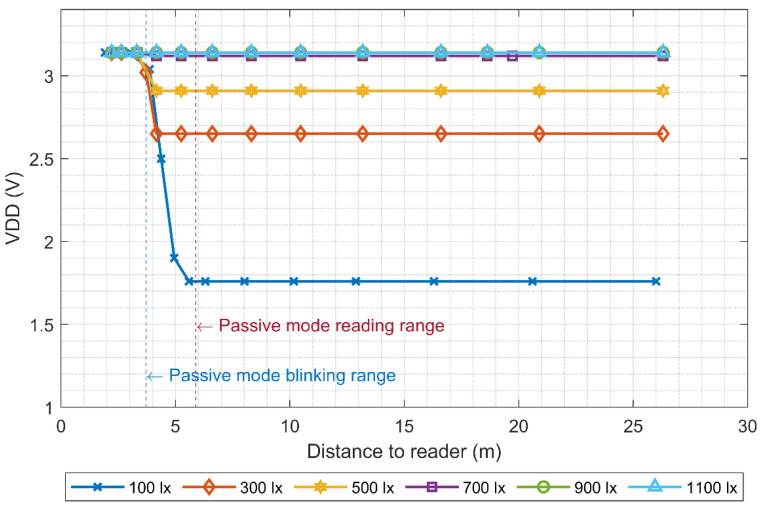
Supply voltage of the RFID tag as a function of the distance to the reader, for various illuminance levels.

To better understand these results, it is helpful to also look at the supply voltage of the tag as a function of illuminance and distance, as shown in Figure 15. By analyzing this graph together with Figure 14, it is possible to observe that the minimum voltage needed to perform a reading task (1.2–1.3 V) was obtained for all the measured illuminance levels. On the other hand, the minimum supply voltage to make the LED of the tag blink was close to 2.8 V, which is more than double the amount of the reading voltage. However, as can be observed in Figure 8, this voltage dropped considerably while the LED was blinking, due to the forward current that goes through the diode.

Looking at these results, it is valid to think that even though the solar cells might be giving enough power to the tag for illuminance levels below 500 lx, the LED is not able to blink because of the small supply voltage values. The limitation is not due to the available power from the solar cell, but due to the generated output voltage. Therefore, increasing the number of solar cells in series or using solar cells that generate higher output voltages would allow the use of the “pick to light” tag with illuminance levels below 500 lx, making it suitable for low illuminance indoor environments.

It is also important to notice the need of the supply voltage monitoring circuit. As shown, the tag is able to communicate with the reader when the supply voltage is only 1.2 V, but it is only able to blink the LED when the supply voltage is around 2.8 V. Therefore, in order to avoid situations where the “pick to light” tag is not blinking due to the lack of VDD voltage but it is able to communicate with the reader, it is important to share with the reader the status of the supply voltage (VDD), so the reader knows that the tag is not blinking even if it is able to communicate with the tag.

### 3.3. Solar Cells with Boost Regulator

To increase the output voltage provided by the solar cell, the possibility of using a boost regulator was considered. In this configuration, three solar cells were connected in parallel and their output into a boost regulator whose output was finally connected to the VDD pin of the tag. This boost regulator device increases the DC voltage of the solar cell to a higher value at its output, by reducing the output current with some power efficiency losses in exchange. Therefore, it is essential to choose a boost converter with high efficiency for the input power range of our application. Additionally, it is also important to consider the cold-startup of the device. During the startup, the device will drain current from the solar cells until it achieves a certain output voltage. In this mode, the efficiency of the device is quite low. Once that voltage is reached, the boost regulator is set to boost mode and it will obtain as much power of the solar cell as possible by using the maximum power point tracking (MPPT) function. For these measurements, the EVAL-ADP5090 boost regulator from Analog Devices was used [30]. The evaluation board is set by default to a 3.5 V output. For the cold-startup, it sets the input voltage to 0.38 V and requires a minimum input power of 16 μW from the solar cells, which can be obtained with the proposed arrangements in Figure 9 for most indoor lighting environments. The device exits cold-startup when the output voltage is over 1.93 V.

Because it is more practical to exit cold-startup as quickly as possible, the solar cell arrangement previously described was proposed for this setup. In this case, since the boost converter sets its input voltage to a fixed value around 0.38 V, it would be more adequate to arrange the three solar cells in parallel. This way, the input power would be greater during the startup, and equal to the series arrangement once the device enters boost mode.

To measure the new setup, this new cell arrangement was connected directly to the input of the boost converter. Then, the output was connected to the RFID tag, and the maximum supply voltage was measured for different illuminance levels, using a halogen lamp and a digital light meter to control the illuminance level. Besides that, the time it took the output voltage to stabilize or reach its maximum value was also measured, as this parameter is also important to consider before choosing this setup for practical applications. Table 2 shows the measured values, which were taken after discharging the internal capacitors of the boost regulator for each measurement.

As shown, the boost regulator is not able to exit the cold-startup for illuminance values of 300 lx and below. The most plausible explanation is that as the RFID tag has a base supply current of 7 μA, the capacitors at the output of the boost converter are being charged and discharged at the same time. As a result, they are not able to reach the 1.93 V threshold to enter boost mode. Related to this, the efficiency of the converter is lower during the cold-startup, so that is also contributing to the problem. To corroborate this hypothesis, additional measurements were taken. This time, the tag was connected to the boost regulator through a switch, after the converter reached boost mode. Table 3 shows the measured results.

As Table 3 shows, the boost regulator is able to reach the 3.5 V output when the tag is not connected. However, it is worth noting that it takes some time to obtain this voltage value, especially in low illuminance environments. When the tag is connected, the supply voltage drops from 3.5 V to 3.15 V due to the integrated voltage limiter that was introduced in Section 2.2. Nonetheless, the supply voltage does not decrease any further with the course of time. It is also shown that the time to reach the maximum output voltage is generally reduced if the tag is connected afterwards.

**Figure 16:**
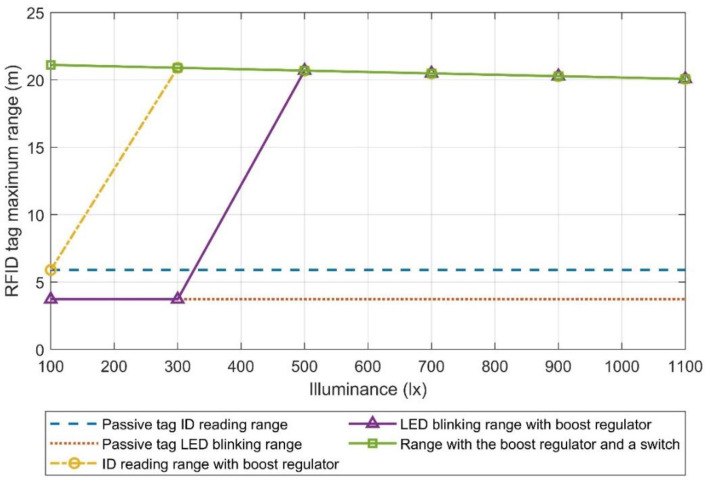
Maximum tag ID reading range and LED blinking range of the RFID tag with the photovoltaic harvesting system proposed in Figure 9b, when connecting the tag directly to the output of the boost regulator and when doing it through a switch.

Figure 16 shows the maximum range of the RFID tag with the arrangement of three solar cells in parallel with a boost regulator. If a switch is used to connect the tag to the output of the boost regulator after exiting cold-startup, both the tag ID reading and the LED blinking functions have a range of 21 m for illuminance values as low as 100 lx. That means that this arrangement has a longer LED blinking range compared to the one in which the solar cells where connected in series, for illuminance values below 500 lx, but at the expense of waiting a long time until maximum output voltage is reached. On the other hand, if the tag is connected directly to the output of the boost regulator without any switch, the LED blinking range is similar to the one when the solar cells are connected in series.

Considering these results, both arrangements have a similar range, unless a switch is used to connect the tag after boost mode is reached, which considerably increases the range of the second arrangement. However, the time it takes the boost regulator to exit cold-startup may make that arrangement impractical in some situations. It is also worthwhile to mention that if a switch is used along with the boost regulator, the results obtained in Figure 16 could also be achieved using a serial connection of the photovoltaic cells, due to the MPPT feature of the boost regulator. Nevertheless, using a serial connection would greatly increase the time to exit cold-startup, due to its lower output current at a fixed output voltage of 380 mV.

Table 4 shows a comparison of the obtained results against other implementations of the state-of-the-art. Although direct comparison is sometimes difficult since other implementations use other light sources such as outdoor sunlight or different required load conditions for supply and power consumption, the reported results for illuminance levels as low as 100 lx are in the state of the art.

Table 5 shows the cost of both implementations. The total cost is EUR 45.5 for the first arrangement and EUR 49.76 for the second. As can be observed, most of the cost is dominated by the tag itself. The cost of the tag can be reduced significantly when produced massively.

Finally, notice that regardless of the arrangement employed, it is necessary to use a VDD monitoring system for “pick to light” application, which is something that the implemented RFID tag contains (Section 2.3). With this system, the tag sends a response that will tell the reader if the available power is enough to make the LED of the tag blink.

## 4. Discussion

A novel photovoltaic cell-powered RFID tag for indoor “pick to light” applications is presented. Firstly, the lighting conditions of various indoor environments were analyzed, and a specific photovoltaic harvester was chosen. It was concluded that besides the illuminance level of the environment, it is crucial to consider the SPD of the light source and the spectral efficiency of the photovoltaic harvester for an optimum performance. The combination of a solar cell plus a halogen bulb as a light source offers the highest harvested power for the same illuminance level compared to other light source and light harvester types. As explained before, the luminance level which characterizes the spectral power within the visible spectrum (380–750 nm) does not consider the energy in the infrared region. Halogen bulbs, as explained in Section 2.1, present a large amount of energy in the infrared region at the expense of higher power consumption when compared to fluorescents or LEDs. This energy in the infrared region is effectively harvested by the solar cell generating higher output power and open circuit voltage.

A maximum output power of 400 μW at 2 V output voltage was obtained with an illuminance level of only 500 lx using three solar cells in series and a halogen bulb. Next, the architecture of the implemented “pick to light” RFID tag was presented. As the large input power range of the harvesting system can cause strong variations in the supply voltage, a novel voltage limiter architecture was presented to minimize supply changes against process and temperature variations. Additionally, a supply voltage monitoring circuit was found to be essential for a “pick to light” application, as it will tell the RFID reader if there is enough supply voltage to turn on the RFID tag’s LED.

Finally, two different photovoltaic cell arrangements were proposed to power the “pick to light” tag and their performance was characterized. The first arrangement of three solar cells in series improved the LED blinking range of the tag from 3.7 to 21 m for illuminance values above 500 lx. However, despite having enough available power, the LED blinking range did not improve for illuminance levels below 500 lx due to the limited output voltage of the photovoltaic cells. A second arrangement was proposed to increase the output voltage of the photovoltaic energy harvesting system, which included three solar cells in parallel that were connected to a boost regulator, whose output was connected to the VDD pin of the tag. The main disadvantage of using a boost regulator is that it needs to exit cold-startup before entering boost mode, which may take a long time. This second arrangement showed similar results to the first one due to the base supply current of the RFID tag, which does not allow the boost regulator to exit cold-startup for illuminance values below 500 lx. In this situation (illuminance level of 500 lx), the system needs to wait 21 s from the moment the tag is illuminated until enough energy is harvested and the boost regulator exits cold-startup and the tag can start blinking. As a result, the second arrangement was modified by inserting a switch in the connection between the output of the boost regulator and the RFID tag. The switch is open while the boost regulator is in cold-startup and closed when it reaches boost mode. With this modification, the LED blinking range of the tag grew to 21 m at illuminance levels as low as 100 lx, which is a significantly better result than that obtained with the previous arrangements. Nevertheless, this arrangement is more complex and requires some time to exit cold-startup, which might be impractical in some situations. As an example, for an illuminance level of 100 lx, the required waiting time is 75 s from the moment the tag is illuminated until enough energy is harvested, so that the boost regulator exits cold-startup and the tag can start blinking. For an illuminance level of 500 lx, this time is reduced to only 12 s.

To obtain those results but without the mentioned disadvantages (waiting time), the first arrangement could be modified by increasing the number of solar cells or using solar cells that generate higher output voltages.

## 5. Patents

Beriain, A.; Zalbide, I.; Jimenez, A.; Galarraga, I.; Berenguer, R.; Alonso, A.; Navarro, E. A Radio Frequency Identification (RFID) tag and a method for limiting supply voltage of a RFID tag. 22 April 2016. Patent: EP 3236392 B1, US 20190130237 A1.

## Figures and Tables

**Table 1:** Power output comparison of potential devices to power the RFID tag.

Device	Type	Package Area (mm^2^)	Active Area (mm^2^)	Peak Power 22 klx (μW)	Peak Power 500 lx (μW)	Power Per Package Area 22 klx (μW/mm^2^)	Power Per Package Area 500 lx (μW/mm^2^)
PDB-C160SM [21]	Photodiode	16.85	8	40	0.9	2.37	0.05
PD70-01C/TR7 [17]	Photodiode	25	8	45	1.1	1.8	0.04
SFH 2704 [18]	Photodiode	3.6	1	15	0.3	4.17	0.08
PDB-C171SM [19]	Photodiode	21.2	8	63	1.35	2.97	0.06
SLSD-71N300 [22]	Photodiode	50.8	50	20	0.8	0.39	0.02
KXOB25-05X3F [20]	Solar cell	184	138	>1126	11.57	6.12	0.06

**Table 2:** Relevant parameters measured for the three photovoltaic cells in parallel with boost regulator setup.

Illuminance (lx)	Input Current during Startup (μA)	Input Voltage during Startup (mV)	Solar Cell Open Circuit Voltage (V)	Maximum Output Voltage (V)	Time to Reach Maximum Output Voltage (s)
100	160	376.6	0.683	0.735	42
300	656	380.5	0.893	1.722	120
500	810	383.3	0.97	3.153	20
700	1060	385.3	1.05	3.153	13
900	1450	389.5	1.13	3.153	8
1100	1780	391	1.18	3.153	6

**Table 3:** Measurements without connecting the tag until the boost converter exits cold-startup.

Illuminance (lx)	Maximum Output Voltage without Tag (V)	Time to Reach Maximum Output Voltage (s)	Output Voltage after Connecting the Tag (V)
100	3.5	75	3.149
300	3.5	14	3.152
500	3.5	12	3.153
700	3.5	10	3.153
900	3.5	7	3.153
1100	3.5	4	3.153

**Table 4:** State-of-art (SoA) of RFID sensors and actuators with a solar harvester.

	[33,34]	[10]	[7]	This Work	
				With Schottky Diode	With Boost Regulator
IC read sensitivity	−8.3 dBm (passive) −31 dBm (BAP)	−7 dBm (passive) −15 dBm (BAP)	−17 dBm (passive) −24 dBm (BAP)	−14 dBm (passive) −24 dBm (BAP) −35 dBm (Enhanced BAP)	
PV harv. area	2.8 cm^2^		54.02 cm^2^	5.52 cm^2^	
Maximum range	4 m @ 925 MHz	7.3 m @ 866 MHz	27 m @ 932 MHz	20.3 m @ 868 MHz	21 m @ 868 MHz
Tag Sensitivity			−25.34 dBm @ 932 MHz	−23.34 dBm @ 868 MHz	−23.68 dBm @ 868 MHz
Lighting conditions	1 sun (solar simulator)	500–1000 lx		500 lx (halogen)	100 lx (halogen)
Power load	7.5 μW (read) 20 μW (20000 meas./h)	9.6 μW (read) 270 μW (single temp. read)	45 μW (read)	8 μW (read) 31 μW (blink)	
Tag features	Temperature sensor	Temperature sensor	Temperature sensor, MCU and humidity sensor	LED indicator	

**Table 5:** Cost comparison (prices from Digi-Key or manufacturer website).

Component	Unit Price @ One Unit	Unit Price @ 1000 Units	Quantity (First Arrangement)	Quantity (Second Arrangement)
EVAL01-Stella-R-DKWB	40 EUR	-----	1	1
KXOB25-05X3F	1.77 EUR	1.20086 EUR	3	3
RB751V40,115	0.19 EUR	0.03043 EUR	1	0
ADP5090	4.45 EUR	2.69662 EUR	0	1
